# NudE regulates dynein at kinetochores but is dispensable for other dynein functions in the *C. elegans* early embryo

**DOI:** 10.1242/jcs.212159

**Published:** 2018-01-01

**Authors:** Patrícia A. Simões, Ricardo Celestino, Ana X. Carvalho, Reto Gassmann

**Affiliations:** 1 Instituto de Biologia Molecular e Celular, Universidade do Porto, 4200-135 Porto, Portugal; 2 Instituto de Investigação e Inovação em Saúde (i3S), Universidade do Porto, 4200-135 Porto, Portugal

**Keywords:** Dynein, Kinetochore, NudE, NDE1, Lis1, PAFAH1B1, RZZ, Spindle assembly checkpoint

## Abstract

In mitosis, the molecular motor dynein is recruited to kinetochores by the Rod–Zw10–Zwilch complex (RZZ) and Spindly to control spindle assembly checkpoint (SAC) signaling and microtubule attachment. How the ubiquitous dynein co-factors Lis1 and NudE contribute to these functions remains poorly understood. Here, we show that the *C. elegans* NudE homolog NUD-2 is dispensable for dynein- and LIS-1-dependent mitotic spindle assembly in the zygote. This facilitates functional characterization of kinetochore-localized NUD-2, which is recruited by the CENP-F-like proteins HCP-1 and HCP-2 independently of RZZ–Spindly and dynein–LIS-1. Kinetochore dynein levels are reduced in *Δnud-2* embryos, and, as occurs upon RZZ inhibition, loss of NUD-2 delays the formation of load-bearing kinetochore–microtubule attachments and causes chromatin bridges in anaphase. Survival of *Δnud-2* embryos requires a functional SAC, and kinetochores without NUD-2 recruit an excess of SAC proteins. Consistent with this, SAC signaling in early *Δnud-2* embryos extends mitotic duration and prevents high rates of chromosome mis-segregation. Our results reveal that both NUD-2 and RZZ–Spindly are essential for dynein function at kinetochores, and that the gain in SAC strength during early embryonic development is relevant under conditions that mildly perturb mitosis.

## Abstract (precis)

**Highlighted Article:** The sole *C. elegans* homolog of NudE and NudEL, ubiquitous co-factors of the molecular motor dynein, contributes to chromosome segregation fidelity in early embryogenesis.

## INTRODUCTION

Accurate segregation of chromosomes during mitosis depends on attachments between spindle microtubules and the kinetochore, a multi-protein assembly that is localized on the centromeric region of each sister chromatid (Musacchio and Desai, 2017). In higher eukaryotes, the mitotic kinetochore is devoid of microtubules at nuclear envelope breakdown (NEBD), so an important first task is to capture microtubules that grow out from the two poles of the nascent spindle. The molecular motor dynein localizes to the kinetochore and has been implicated in establishing initial contacts with the side of spindle microtubules (Alexander and Rieder, 1991; Pfarr et al., 1990; Steuer et al., 1990). The lateral attachments made by dynein are transient and need to be converted into ‘end-on’ attachments, in which dynamic microtubule plus-ends are stably embedded in the outer kinetochore. The end-on attachment configuration, which requires the KNL1, Mis12 complex, Ndc80 complex (KMN) network (Cheeseman et al., 2006; Varma and Salmon, 2013), must be load-bearing to withstand the tension generated when sister kinetochores are correctly connected to opposite spindle poles (bi-orientation) (Salmon and Bloom, 2017).

Because chromosome bi-orientation is a stochastic process, cells use a signaling system called the spindle assembly checkpoint (SAC) to generate a diffusible cell cycle inhibitor at kinetochores that have not yet succeeded in establishing end-on microtubule attachments, thereby preventing premature sister chromatid separation that could lead to chromosome loss (Musacchio, 2015). The core SAC components Mad1 and Mad2 are initially recruited to unattached kinetochores at NEBD to activate SAC signaling, and must be removed once microtubules have attached to silence the SAC (Maldonado and Kapoor, 2011). The microtubule minus-end-directed motor activity of dynein is thought to contribute to SAC silencing by transporting Mad1, Mad2 and other checkpoint proteins away from kinetochores towards spindle poles (referred to as ‘stripping’ or ‘streaming’) (Howell et al., 2001; Wojcik et al., 2001).

Two molecular pathways have been implicated in dynein recruitment to the kinetochore. The first pathway to be discovered consists of the three-subunit Rod–Zw10–Zwilch (RZZ) complex and the coiled-coil protein Spindly (Griffis et al., 2007; Starr et al., 1998). RZZ directly recruits Spindly to kinetochores, and Spindly in turn acts as an adaptor that brings together dynein with its activator dynactin in a tripartite dynein–dynactin–Spindly complex capable of processive motility *in vitro* (Barisic et al., 2010; Chan et al., 2009; Gama et al., 2017; McKenney et al., 2014; Mosalaganti et al., 2017). Consistent with a role for kinetochore dynein in initial microtubule capture, RZZ inhibition in the one-cell *C. elegans* embryo delays the formation of load-bearing attachments between kinetochores and microtubules that oppose strong cortical pulling forces acting on astral microtubules at this stage (Gassmann et al., 2008). In addition to dynein recruitment, RZZ contributes to kinetochore localization of Mad1 and Mad2 (Buffin et al., 2005; Kops et al., 2005), and in *C. elegans* MAD-1/MAD-2 recruitment additionally requires the Spindly homolog SPDL-1 (Gassmann et al., 2008; Yamamoto et al., 2008). RZZ inhibition in *D. melanogaster* and *C. elegans* abrogates SAC signaling and causes chromosome bridges in anaphase, which is indicative of persistent defects in chromosome bi-orientation, and the resulting aneuploidy is lethal (Gassmann et al., 2008; Karess and Glover, 1989; Scaërou et al., 1999; Smith et al., 1985; Starr et al., 1997; Williams and Goldberg, 1994).

A second pathway through which dynein is recruited to the kinetochore involves the paralogs NudE and NudEL (NudE/L; also known as NDE1 and NDEL1 in mammals, respectively). In contrast to RZZ and Spindly, whose interactions with dynein appear to be restricted to the kinetochore, NudE/L are ubiquitous dynein co-factors that contribute to many of the diverse functions of the dynein motor in dividing and non-dividing cells, including organelle transport, positioning of nuclei and centrosomes, and mitotic spindle assembly (Kuijpers et al., 2016; Lam et al., 2010; Liang et al., 2004; Lu and Prehoda, 2013; Ma et al., 2009; Moon et al., 2014; Pandey and Smith, 2011). NudE/L bind directly to both dynein and the dynein co-factor Lis1 (also known in mammals as PAFAH1B1) (Feng et al., 2000; Niethammer et al., 2000; Sasaki et al., 2000; Zylkiewicz et al., 2011), mutations in which cause the brain development disease lissencephaly (Reiner et al., 1993). Consistent with the idea that NudE/L help tether Lis1 to dynein, overexpression of Lis1 in *A. nidulans* and *S. cerevisiae* suppresses the phenotype of NudE/L loss (Efimov, 2003; Li et al., 2005). Lis1 binds to the motor domain of dynein and increases the affinity of dynein for microtubules (Huang et al., 2012; McKenney et al., 2010; Toropova et al., 2014), suggesting that one critical role for NudE/L and Lis1 is to improve the ability of dynein to bear load when transporting large cargo.

NudE/L are recruited to kinetochores through a direct interaction with the coiled-coil protein CENP-F (Liang et al., 2007; Vergnolle and Taylor, 2007). In human cultured cells, NudE/L inhibition achieved through RNAi-mediated depletion, antibody injection and overexpression of dominant-negative fragments reduces dynein levels at kinetochores and causes defects in chromosome congression and segregation (Liang et al., 2007; Raaijmakers et al., 2013; Stehman et al., 2007; Vergnolle and Taylor, 2007; Yan et al., 2003). Similarly, a *D. melanogaster* null allele of the sole NudE/L homolog *nudE* impairs chromosome congression and abolishes dynein-dependent ‘streaming’ of outer kinetochore components, albeit without obvious effects on dynein localization to kinetochores (Wainman et al., 2009). Another characteristic phenotype of NudE/L inhibition in cultured human cells and *D. melanogaster* is mitotic arrest, which likely reflects impaired removal of Mad1/Mad2 from kinetochores and/or persistent SAC activation due to problems in kinetochore–microtubule attachment. While the importance of NudE/L for mitosis is firmly established, the specific contribution of kinetochore-localized NudE/L remains difficult to pin down because NudE/L are required for proper spindle architecture (Raaijmakers et al., 2013; Stehman et al., 2007; Wainman et al., 2009). One interesting unanswered question is how the kinetochore function of NudE/L relates to that of RZZ–Spindly.

Here, we take advantage of a genetic null allele of the sole *C. elegans* NudE/L homolog *nud-2* to investigate its role during mitosis in the early embryo. Our results provide insight into the function of the NUD-2-dependent dynein pathway at kinetochores and illustrate the importance of SAC signaling for the fidelity of chromosome segregation in early embryogenesis.

## RESULTS

### The *C. elegans* NudE/L homolog NUD-2 is dispensable for the dynein-dependent pulling forces that position centrosomes and pronuclei in the zygote

**Fig. 1.**
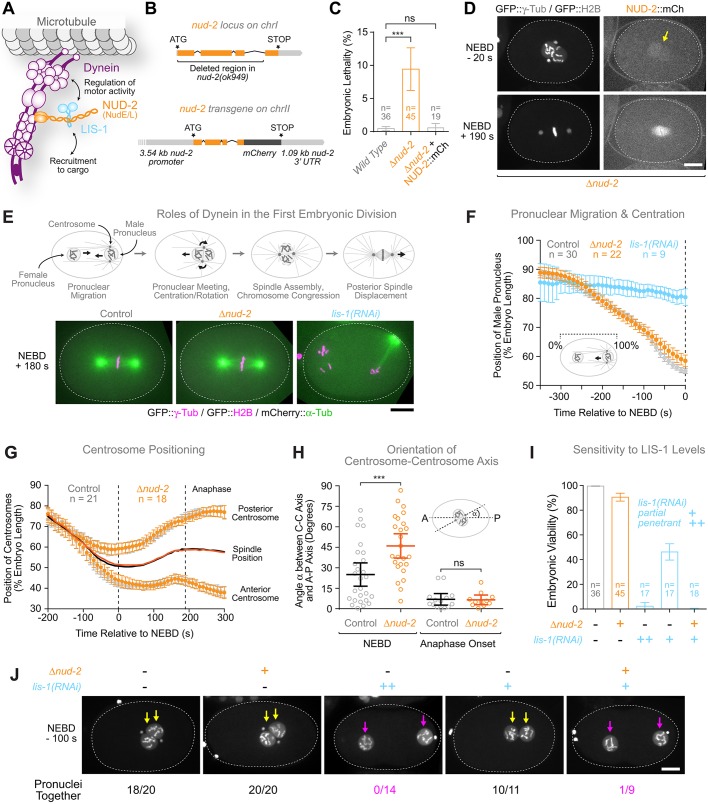
**NUD-2 is dispensable for positioning centrosomes and pronuclei in the *C. elegans* one-cell embryo but reinforces dynein function when LIS-1 levels are reduced.** (A) Schematic summarizing general roles for the dynein co-factors NudE/L and Lis1: regulation of motor activity and recruitment to cargo. (B) Schematic of the null allele *nud-2(ok949)*, referred to as *Δnud-2*, and the *nud-2* transgene used in this study. (C) Graph showing modest embryonic lethality in *Δnud-2*, which is rescued by transgene-encoded NUD-2::mCherry. *n* indicates the number of hermaphrodite mothers whose progeny was counted (>250 total progeny per condition). ***P<0.001; ns, not significant, *P*>0.05 (one-way ANOVA followed by Bonferroni's multiple comparison test). (D) Stills from a time-lapse sequence in the one-cell embryo showing enrichment of NUD-2::mCherry in pronuclei just prior to NEBD (arrow) and on the mitotic spindle in metaphase. A slight enrichment at kinetochores is also visible at metaphase. Scale bar: 10 µm. (E) Top: cartoons highlighting the role of dynein in the positioning of centrosomes and pronuclei, spindle assembly and chromosome congression during the first embryonic division. Black arrows indicate dynein-dependent movement. Bottom: stills from time-lapse sequences showing normal mitotic spindle assembly and positioning in a *Δnud-2* embryo. By contrast, depletion of LIS-1 (a binding partner of NUD-2) results in failure of centrosome separation and pronuclear migration. Scale bar: 10 µm. (F) Graph showing normal migration kinetics of the male pronucleus in *Δnud-2* embryos and failure of pronuclear migration after *lis-1(RNAi)*. The position of the pronucleus along the anterior-posterior axis (see cartoon) was determined in images captured every 10 s. Individual traces were normalized to embryo length, averaged for the indicated number *n* of embryos, and plotted against time. (G) Graph showing the normal kinetics of centrosome positioning along the anterior-posterior axis in *Δnud-2* embryos, plotted as described for F. Solid lines indicate the midpoint between the two centrosomes (spindle position). Anaphase begins at 200 s. (H) Angle between the centrosome–centrosome axis and the anterior–posterior (A-P) axis in one-cell embryos at NEBD and anaphase onset. Circles correspond to measurements in individual embryos. ***P<0.001; ns, not significant, *P*>0.05 (*t*-test). (I) Embryonic lethality assay demonstrating that *Δnud-2* embryos are sensitive to a reduction in LIS-1 levels. (J) Stills from time-lapse sequences in the one-cell embryo showing additive defects in pronuclear migration when LIS-1 is partially depleted in *Δnud-2* embryos. The number of embryos in which the two pronuclei in the posterior half were joined at 100 s prior to NEBD/total number of embryos examined is indicated below the stills. Yellow arrows highlight joined pronuclei and magenta arrows separate pronuclei. Scale bar: 10 µm. The dotted line in D, E and J indicates the periphery of the one-cell embryo. All error bars represent the 95% c.i.

The dynein co-factor Lis1 binds the AAA+ ring of dynein heavy chain to regulate motor activity, and NudE/L are proposed to reinforce the Lis1–dynein interaction by tethering Lis1 to dynein. In addition, Lis1 and NudE/L are involved in dynein recruitment to cargo (Cianfrocco et al., 2015; Vallee et al., 2012) (Fig. 1A). In *C. elegans*, LIS-1 is essential for dynein-dependent processes in the dividing one-cell embryo (Cockell et al., 2004). To ask whether NUD-2, the sole *C. elegans* homolog of NudE/L, also has a role in the early embryo, we took advantage of the existing *nud-2* null allele *ok949* (*Δnud-2*) (Fridolfsson et al., 2010) (Fig. 1B). Homozygous *Δnud-2* embryos exhibited a low, but significant level of lethality (9.4±3.2% versus 0.47±0.29% for wild-type controls; all values in this paper are mean±95% c.i.), which was rescued by transgene-encoded NUD-2::mCherry expressed from the *nud-2* promoter and 3′ untranslated region (0.63±0.61% of lethality) (Fig. 1B,C). Live imaging revealed that NUD-2::mCherry was expressed in the one-cell embryo and localized predominantly to the mitotic spindle, as expected for a dynein-associated factor (Fig. 1D; Movie 1). Unlike dynein, NUD-2::mCherry became enriched in the nucleus just prior to NEBD, which is similar to what has been described for LIS-1 (Cockell et al., 2004), suggesting that the two proteins interact at this stage (Fig. 1D, arrow).

Dynein activity is essential for generating the microtubule-based pulling forces in the cytoplasm and at the cell cortex that position centrosomes and pronuclei in the first embryonic division (Fig. 1E). To precisely define the contribution of LIS-1 and NUD-2 to these processes, we tracked the position of centrosomes and pronuclei over time in embryos co-expressing GFP::histone H2B and GFP::γ-tubulin. Depletion of LIS-1 by RNAi completely inhibited centrosome separation and pronuclear migration, in agreement with prior work (Cockell et al., 2004) (Fig. 1E,F). By contrast, centrosome separation, the migration and centration of pronuclei, spindle assembly and asymmetric spindle positioning occurred with essentially identical kinetics in *Δnud-2* embryos and controls (Fig. 1E–G). The only slight defect in *Δnud-2* embryos was a delay in the orientation of the nucleus–centrosome complex during the centration phase (the angle between centrosome-centrosome and anterior-posterior axis was 46±9° versus 25±9° in controls at NEBD) (Fig. 1H). Spindle orientation subsequently corrected during prometaphase (7±4° versus 7±4° in controls at anaphase onset). We conclude that, in contrast to LIS-1, NUD-2 is largely dispensable for the dynein-dependent pulling forces that position centrosomes and pronuclei during the first embryonic division.

### NUD-2 supports dynein function when LIS-1 levels are reduced

In *X. laevis* egg extracts (Wang and Zheng, 2011), *A. nidulans* (Efimov, 2003) and *S. cerevisiae* (Li et al., 2005), defects after NudE/L inhibition can be suppressed by providing an excess of Lis1. We therefore asked whether the importance of NUD-2 for dynein function was modulated by LIS-1 levels in the embryo. Penetrant depletion of LIS-1 (see Materials and Methods) was fully lethal for the progeny of mothers injected with dsRNA (2±3% viable embryos), but 46±7% of embryos survived a partial decrease in LIS-1 levels achieved by shorter RNAi treatment [partial *lis-1(RNAi)*] (Fig. 1I). By contrast, partial *lis-1(RNAi)* was not tolerated in *Δnud-2* embryos (0±1% viability). In agreement with the effect on embryonic viability, we found that pronuclear migration in *Δnud-2* embryos showed an enhanced sensitivity to a partial decrease in LIS-1 levels (Fig. 1J). In control embryos, *Δnud-2* embryos and partial *lis-1(RNAi)* embryos, the two pronuclei were together in the posterior half at 100 s prior to NEBD. By contrast, pronuclei had failed to meet by the same time point in *Δnud-*2 embryos after partial *lis-1(RNAi)*, which is similar to the situation in penetrant *lis-1(RNAi)* embryos. We conclude that the contribution of NUD-2 to dynein-dependent pronuclear migration becomes important when LIS-1 levels are reduced.

### NUD-2 is required for accurate chromosome segregation

**Fig. 2.**
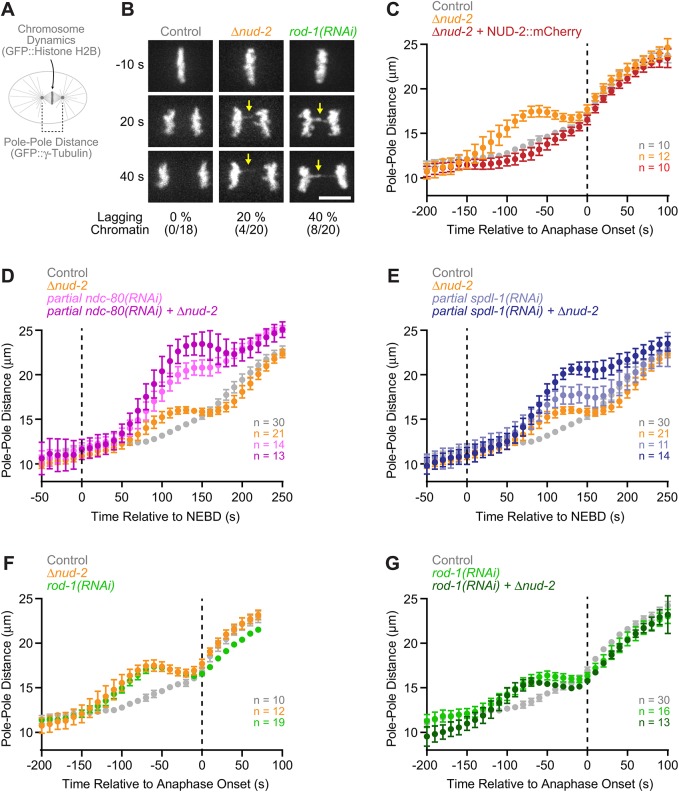
**NUD-2 loss and RZZ inhibition cause identical delays in the formation of load-bearing kinetochore-microtubule attachments.** (A) Assays for kinetochore function in the one-cell embryo. The distance between spindle poles serves as a readout for the ability of kinetochores to form load-bearing attachments to microtubules. (B) Stills from time-lapse sequences in one-cell embryos expressing GFP::histone H2B, showing that loss of NUD-2 results in lagging anaphase chromatin (arrows), similar to what is observed upon depletion of the RZZ subunit ROD-1. The number of embryos in which lagging anaphase chromatin was observed/total number of embryos examined is indicated below the stills. Scale bar: 5 µm. (C–G) Plots of spindle pole separation kinetics in one-cell embryos expressing GFP::γ-tubulin, showing that loss of NUD-2 and ROD-1 depletion results in identical defects. Pole–pole distances were measured in images acquired every 10 s, averaged for the indicated number *n* of embryos, and plotted against time. Error bars represent the 95% c.i.

Despite normal spindle assembly and positioning (Fig. 1E,G), close inspection of chromosome dynamics revealed that loss of NUD-2 compromised the fidelity of chromosome segregation (Fig. 2A,B). In four out of 20 one-cell embryos, we observed lagging chromatin in anaphase, which was never observed in 18 control embryos. This was unlikely to be an indirect consequence of the modest spindle orientation delay, since mutants of *dnc-1* (the *C. elegans* dynactin p150), which exhibit significantly more pronounced spindle mis-orientation, do not show chromosome segregation defects (Barbosa et al., 2017). The anaphase chromosome bridges in *Δnud-2* embryos were reminiscent of those observed after inhibition of RZZ (Gassmann et al., 2008), although the defect was slightly more frequent for *rod-1(RNAi)* (eight out of 20 embryos) and chromosome bridges tended to be more pronounced in these embryos (Fig. 2B). These results suggested that NUD-2, like RZZ, plays a role in kinetochore–microtubule attachment.

### Loss of NUD-2 and RZZ inhibition cause an identical delay in the formation of load-bearing kinetochore–microtubule attachments

To define how NUD-2 contributes to kinetochore function, we determined the kinetics of spindle pole separation in the one-cell embryo (Fig. 2A), where attachments between kinetochores and spindle microtubules counteract cortical forces that pull on astral microtubules for asymmetric positioning of the spindle. Premature pole separation prior to anaphase onset is therefore indicative of defects in the formation of kinetochore–microtubule attachments that can sustain tension (‘load-bearing’ attachments) (Cheeseman et al., 2004; Desai et al., 2003; Gassmann et al., 2008; Oegema et al., 2001). In *Δnud-2* embryos, the pole–pole distance increased prematurely following NEBD, started to recover around 100 s after NEBD and had become identical to controls at the time of sister chromatid separation (anaphase onset) (Fig. 2C–F). Transgene-encoded NUD-2::mCherry rescued premature pole separation in *Δnud-2* embryos, demonstrating that the defect was caused by loss of NUD-2 (Fig. 2C). These results argue that NUD-2 is not per se required for load-bearing kinetochore–microtubule attachments but that NUD-2 accelerates their formation, as previously shown for RZZ (Gassmann et al., 2008). We observed additive defects when the core microtubule attachment site was compromised in *Δnud-2* embryos through partial depletion of NDC-80 or SPDL-1 (which results in RZZ-mediated inhibition of NDC-80; Cheerambathur et al., 2013; Gassmann et al., 2008) (Fig. 2D,E). This is consistent with the idea that NUD-2 ensures the timely formation of stable kinetochore–microtubule attachments mediated by the KMN network. Strikingly, the pole separation profile in *Δnud-2* embryos was identical to the profile obtained in *rod-1(RNAi)* embryos (Fig. 2F). Furthermore, depletion of ROD-1 in *Δnud-2* embryos did not exacerbate premature pole separation (Fig. 2G), nor did it increase the fraction of one-cell embryos with lagging anaphase chromatin compared to *rod-1(RNAi)* on its own (six out of 13, and 12 out of 25 embryos, respectively). This indicates that NUD-2 and RZZ act through the same pathway.

In conclusion, *Δnud-2* and *rod-1(RNAi)* result in lagging anaphase chromatin and cause an identical delay in the formation of load-bearing attachments between kinetochores and microtubules. Overall, the phenotypic similarity to that seen upon RZZ inhibition suggests that loss of NUD-2 in the one-cell embryo primarily compromises dynein function at kinetochores, while other dynein-dependent processes remain unaffected.

### NUD-2 localizes to kinetochores and contributes to dynein recruitment

**Fig. 3.**
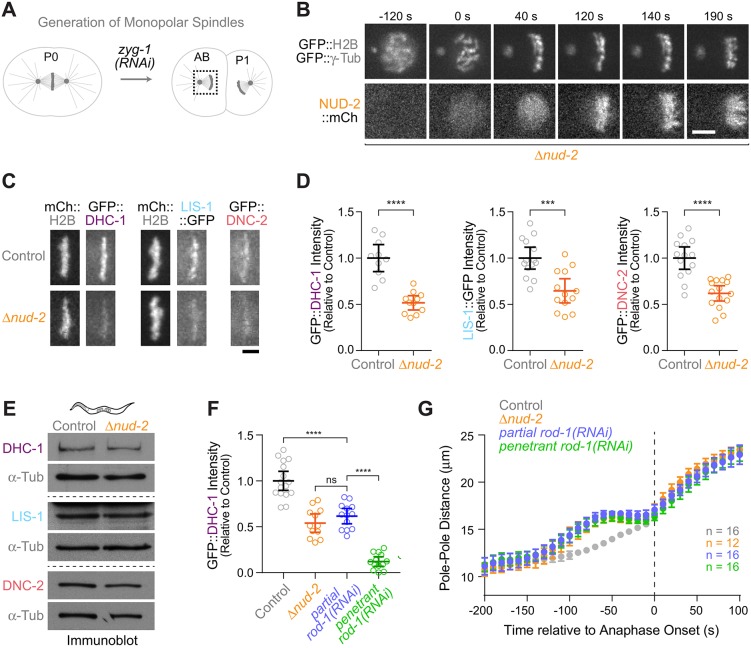
**Kinetochores are sensitive to reduced dynein levels.** (A) Strategy for the generation of monopolar spindles in the second embryonic division. ZYG-1 kinase is required for centriole duplication. In *zyg-1(RNAi)* embryos, the first division is normal, because two intact centrioles are contributed by sperm that is not affected by RNAi. Centrioles are unable to duplicate, resulting in a monopolar spindle in the subsequent division. (B) Stills from a time-lapse sequence in a monopolar AB cell, showing that NUD-2::mCherry is enriched at kinetochores. Scale bar: 5 µm. (C) Stills from time-lapse sequences in monopolar AB cells, showing that loss of NUD-2 decreases kinetochore levels of GFP::DHC-1, LIS-1::GFP, and GFP::DNC-2. Scale bar: 2.5 µm. (D) Quantification of GFP levels on monopolar chromosomes as shown in C, as determined by fluorescence intensity measurements. Circles correspond to measurements in individual embryos. ***P<0.001; ****P<0.0001 (*t*-test). (E) Immunoblots of adult animals, showing that loss of NUD-2 does not decrease total protein levels of DHC-1, LIS-1 or DNC-2. α-Tubulin was used as a loading control. (F) Quantification of GFP::DHC-1 levels on monopolar chromosomes, showing that the reduction in dynein levels after NUD-2 loss can be recapitulated by partial ROD-1 depletion. Circles correspond to measurements in individual embryos. ****P<0.0001; ns, not significant, *P*>0.05 (one-way ANOVA followed by Bonferroni's multiple comparison test). (G) Plots of spindle pole separation kinetics in one-cell embryos expressing GFP::γ-tubulin, displayed as described in Fig. 2C–G. RNAi conditions were identical to those used for the intensity measurements in F. All error bars represent the 95% c.i.

To test whether NUD-2 itself localized to kinetochores, we generated monopolar spindles in the second embryonic division, which facilitates visualization of kinetochore dynein and associated factors (Gassmann et al., 2008) (Fig. 3A). This assay revealed that, in addition to its prominent localization on the spindle, NUD-2::mCherry is enriched at kinetochores (Fig. 3B). We then used the monopolar spindle assay to ask whether NUD-2 is involved in kinetochore recruitment of dynein, LIS-1 and dynactin. Fluorescence intensity measurements revealed that kinetochore levels of GFP::DHC-1 (the *C. elegans* dynein heavy chain), LIS-1::GFP and GFP::DNC-2 (the *C. elegans* dynactin p50) were reduced in *Δnud-2* embryos to 52±8%, 65±13%, and 62±8% of controls, respectively (Fig. 3C,D). Importantly, immunoblotting for DHC-1, LIS-1 and DNC-2 revealed that their total protein levels were unchanged in *Δnud-2* animals (Fig. 3E). We conclude that kinetochore-localized NUD-2 contributes to the recruitment of dynein and its co-factors LIS-1 and dynactin.

### Kinetochores are sensitive to a reduction in dynein levels

The observation that loss of NUD-2 mimicked RZZ inhibition in the pole tracking assay was surprising, since *Δnud-2* embryos still retained >50% of dynein at kinetochores, while RZZ inhibition essentially eliminates dynein from kinetochores (Gassmann et al., 2008). This raised the possibility that kinetochores might be particularly sensitive to a reduction in dynein levels. To test this idea, we partially depleted RZZ to reduce, but not eliminate, kinetochore dynein. Quantification of GFP::DHC-1 intensity at monopolar kinetochores showed that partial ROD-1 depletion reduced GFP::DHC-1 levels to 62±8% of controls, which was similar to kinetochore levels of GFP::DHC-1 measured in *Δnud-2* embryos under the same conditions in parallel experiments (54±10%) (Fig. 3F). By contrast, penetrant depletion of ROD-1 reduced GFP::DHC-1 localization to kinetochores to 12±5%. When we tracked spindle poles in the one-cell embryo under these RNAi conditions, the pole separation profile after partial *rod-1(RNAi)* was indistinguishable from the profile after penetrant *rod-1(RNAi)* and from the profile in *Δnud-2* embryos (Fig. 3G). Thus, conditions that result in a ∼40% reduction in kinetochore dynein levels on monopolar spindles are sufficient to functionally mimic complete loss of kinetochore dynein in an assay that monitors the formation of load-bearing microtubule attachments. While these results do not exclude the possibility that NUD-2 loss directly affects the ability of dynein to hold on to microtubules under load, the implications are that, without NUD-2, dynein levels at the kinetochore are below the threshold required for a productive contribution to microtubule attachment.

### Embryos without NUD-2 depend on a functional SAC for survival

**Fig. 4.**
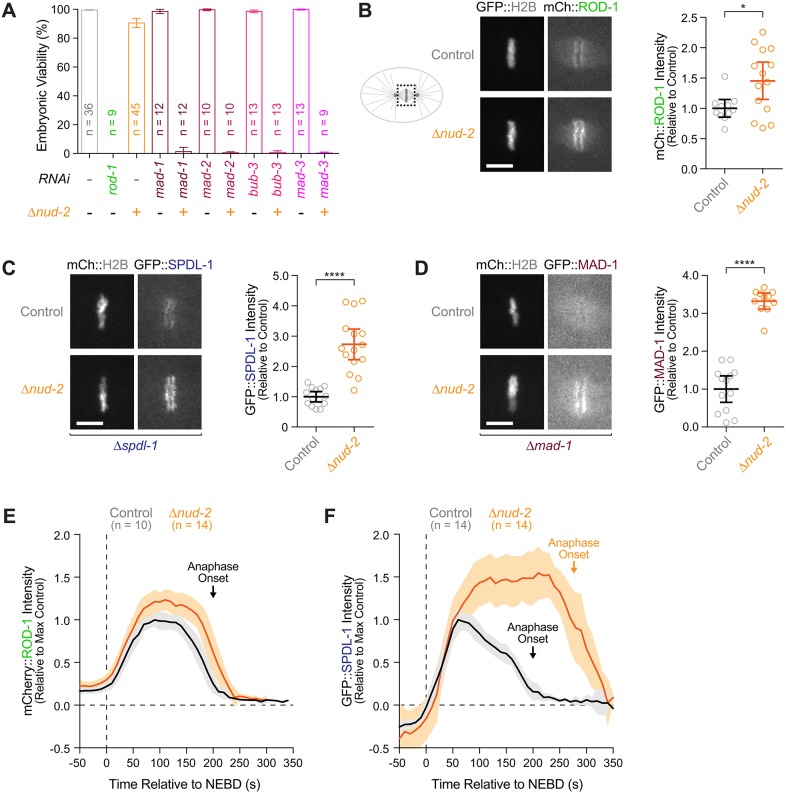
**Embryos without NUD-2 require the SAC for survival and hyper-accumulate SAC components at kinetochores.** (A) Embryonic viability assay demonstrating that NUD-2 loss is lethal when combined with depletion of SAC components. *n* indicates the number of hermaphrodite mothers whose progeny was counted (>250 total progeny per condition). (B–D) Left: stills from time-lapse sequences in the metaphase one-cell embryo showing that NUD-2 loss results in increased kinetochore levels of mCherry::ROD-1 (B), GFP::SPDL-1 (C) and GFP::MAD-1 (D). Right: quantification of mCherry/GFP levels on chromosomes as shown on the left as determined using fluorescence intensity measurements. Circles correspond to measurements in individual embryos. Error bars in A–D represent the 95% c.i. *P<0.05; ****P<0.0001 (*t*-test). (E,F) Quantification of the mCherry::ROD-1 (E) and GFP::SPDL-1 (F) signal on mitotic chromosomes over time in one-cell embryos. Fluorescence intensities were measured in images acquired every 10 s, averaged for the indicated number *n* of embryos, and plotted against time. Values are plotted mean±95% c.i., normalized to the maximum signal in controls.

RZZ inhibition and loss of NUD-2 caused an identical delay in the formation of kinetochore–microtubule attachments and compromised the fidelity of chromosome segregation (Fig. 2), yet the effects on embryonic viability were strikingly different. RZZ inhibition resulted in 100% embryonic lethality, whereas *Δnud-2* embryos were >90% viable (Fig. 4A). In addition, *Δnud-2* animals developed normally and had the same number of progeny as wild-type controls (data not shown). One major difference between RZZ and NUD-2 is that RZZ, in addition to dynein recruitment, also contributes to SAC signaling by recruiting the checkpoint proteins MAD-1 and MAD-2 to kinetochores (Essex et al., 2009). We therefore sought to explore the functional relationship between the SAC and NUD-2. RNAi-mediated depletion of MAD-1 or MAD-2 on their own did not affect the viability of embryos derived from injected mothers (99±2% and 100±1% viability, respectively). By contrast, depletion of MAD-1 or MAD-2 in *Δnud-2* animals reduced embryonic viability from 91±3% to 1±3% and 0±1%, respectively (Fig. 4A). The same synthetic lethality was observed after depletion of two additional SAC proteins, MAD-3 and BUB-3 (Fig. 4A). We conclude that inactivation of the SAC in *Δnud-2* embryos recapitulates the penetrant embryonic lethality of RZZ inhibitions.

### Kinetochores without NUD-2 recruit an excess of SAC proteins

The dependence of *Δnud-2* embryos on a functional SAC suggested that the delay in the formation of kinetochore–microtubule attachments activated the SAC, and that SAC signaling was required to attenuate errors in chromosome segregation. To explore this idea, we first assessed whether localization of checkpoint proteins was altered in *Δnud-2* embryos. By measuring fluorescence intensity, we quantified levels of checkpoint proteins on chromosomes in one-cell embryos expressing transgene-encoded mCherry::ROD-1, GFP::SPDL-1 or GFP::MAD-1. GFP::SPDL-1 and GFP::MAD-1 were expressed in an *spdl-1* and *mad-1* null background, respectively (Moyle et al., 2014; Yamamoto et al., 2008). In *Δnud-2* embryos, kinetochore levels of mCherry::ROD-1, GFP::SPDL-1 and GFP::MAD-1 at metaphase (∼160 s after NEBD) were increased to 145±31%, 273±51%, and 333±21% of control levels, respectively (Fig. 4B–D; Movies 2–4). Thus, lack of NUD-2 results in significantly elevated levels of checkpoint proteins at kinetochores.

In cultured human cells and *D. melanogaster*, dynein contributes to the removal of checkpoint proteins from kinetochores via microtubule-based transport towards spindle poles (Howell et al., 2001; Wojcik et al., 2001). It is not clear whether an analogous mechanism for SAC protein removal from kinetochores operates in *C. elegans*, as we did not observe poleward streaming of SAC proteins or dynein during chromosome congression or metaphase in control embryos. Nevertheless, we sought to address whether the elevated SAC protein levels at metaphase kinetochores of *Δnud-2* embryos could be explained by a failure in dynein-mediated removal of SAC proteins, that is, a defect in SAC silencing. We therefore monitored the levels of mCherry::ROD-1 and GFP::SPDL-1 on chromosomes throughout mitosis (a high nuclear signal precluded a similar analysis for GFP::MAD-1; see Materials and Methods). During the 200-s interval between NEBD (0 s) and sister chromatid separation (anaphase onset; 200 s), kinetochore levels of mCherry::ROD-1 and GFP::SPDL-1 followed a stereotypical profile (Fig. 4E,F). For GFP::SPDL-1, three distinct phases could be distinguished: a recruitment phase (0–70 s), during which kinetochore levels increased rapidly following NEBD, a phase of slow decrease (80–140 s), and a short plateau phase followed by accelerated decrease (150–240 s), during which levels dropped to ∼30% of the peak levels at anaphase onset and became undetectable shortly thereafter (Fig. S1). Correspondingly, mitotic chromosome behavior could be divided into initial poleward and anti-poleward movements (0–70 s), congression to the spindle center (80–140 s), and tight metaphase plate formation (150–200 s) (Fig. S1). After a similar recruitment phase, mCherry::ROD-1 levels on chromosomes initially decreased more slowly than did GFP::SPDL-1 levels but also dropped to ∼30% of peak levels by anaphase onset (Fig. 4E). We conclude that kinetochore levels of RZZ and SPDL-1 are inversely correlated with the formation of load-bearing kinetochore–microtubule attachments. In *Δnud-2* embryos, mCherry::ROD-1 and GFP::SPDL-1 accumulated at early prometaphase kinetochores beyond the peak levels reached in controls, and levels remained elevated until anaphase onset (Fig. 4E,F). The hyper-accumulation in early prometaphase was particularly striking for GFP::SPDL-1. These results argue that rather than just impairing the removal of checkpoint proteins from microtubule-attached kinetochores (SAC silencing), loss of NUD-2 results in increased SAC signaling during prometaphase, which is consistent with the observed delay in the formation of load-bearing kinetochore–microtubule attachments.

### SAC signaling is critical to reduce the rate of chromosome mis-segregation in early *Δnud-2* embryos

**Fig. 5.**
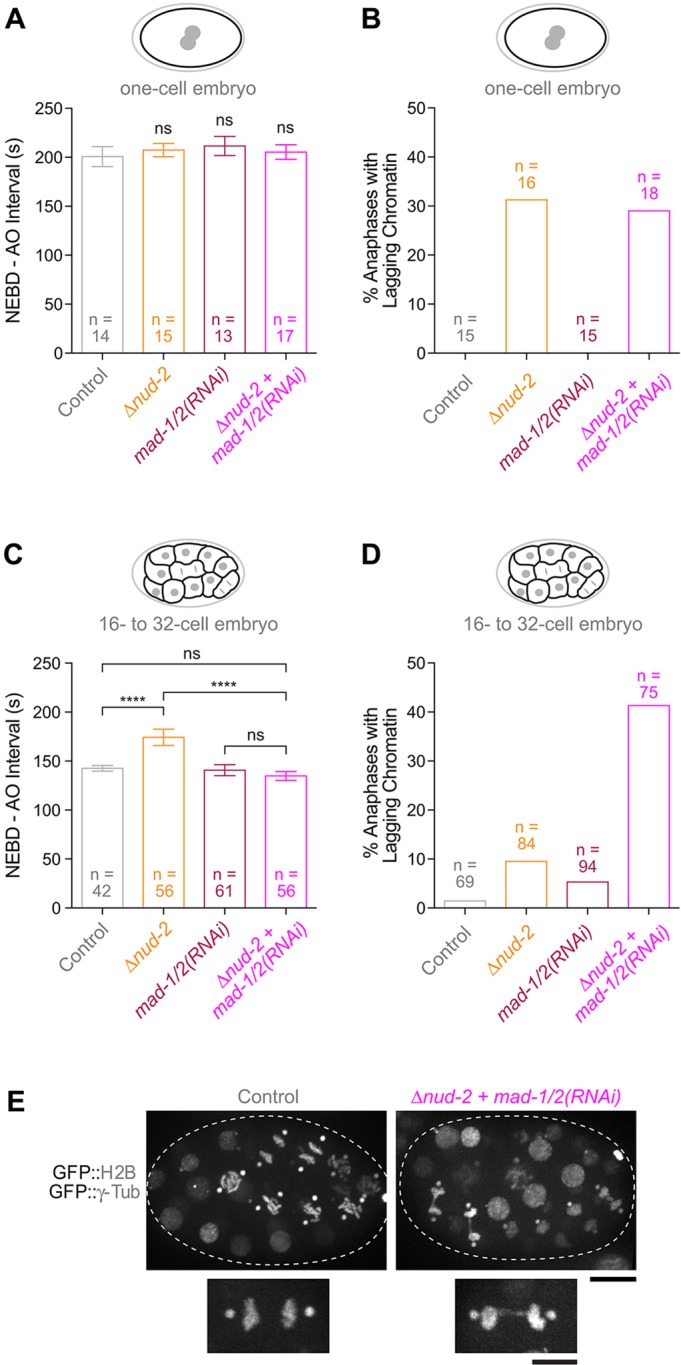
**SAC signaling in early multicellular *Δnud-2* embryos reduces the rate of chromosome mis-segregation.** (A) Interval between NEBD and anaphase onset (AO) in one-cell embryos, showing that loss of NUD-2 has no effect on mitotic timing. *n* indicates the number of embryos. ns, not significant, *P*>0.05 (one-way ANOVA followed by Bonferroni's multiple comparison test). (B) Frequency of anaphases with lagging chromatin in one-cell embryos, showing that SAC inhibition in *Δnud-2* embryos has no additive effect on the chromosome segregation fidelity. (C) Mitotic timing in *Δnud-2* embryos increases at the 16- to 32-cell stage in an SAC-dependent manner. The total number of cells (*n*) scored in at least eight different embryos is indicated. ****P<0.0001; ns, not significant, *P*>0.05 (one-way ANOVA followed by Bonferroni's multiple comparison test). (D) Frequency of anaphases with lagging chromatin at the 16- to 32-cell stage, showing increased rates of chromosome mis-segregation after SAC inhibition in *Δnud-2* embryos. The total number of cells (*n*) scored in at least eight different embryos is indicated. (E) Stills from time-lapse sequences in embryos (dotted line) at the 32-cell stage showing lagging anaphase chromatin in *Δnud-2* embryos after SAC inhibition. Scale bar, 10 µm; inset, 5 µm. All error bars represent the 95% c.i.

To assess whether SAC signaling was strong enough to prolong mitosis in early *Δnud-2* embryos, we determined the interval between NEBD and sister chromatid separation (anaphase onset) in embryos expressing GFP::histone H2B. In one-cell *Δnud-2* embryos, the NEBD–anaphase onset interval was not significantly different from that of controls (207±7 s vs 199±10 s, respectively), nor did co-depletion of NUD-2 with MAD-1 and MAD-2 [*mad-1/2(RNAi)*] shorten the duration of mitosis (Fig. 5A). Thus, despite enhanced recruitment of checkpoint proteins, the strength of the SAC signal is too weak in one-cell *Δnud-2* embryos to delay the cell cycle (with the notable exception of one-cell *Δnud-2* embryos expressing GFP::SPDL-1, which for reasons that are currently unclear delay anaphase onset by ∼70 s relative to controls; Fig. 4F). In agreement with the invariant mitotic duration, co-depletion of NUD-2 with MAD-1 and MAD-2 in one-cell *Δnud-2* embryos did not increase the frequency of lagging anaphase chromatin (29% versus 31% in *Δnud-2* alone) (Fig. 5B).

A recent study, in which maximal SAC activation was triggered at different stages of embryogenesis by addition of a microtubule-depolymerizing drug, demonstrated that SAC signaling becomes more robust in multicellular embryos in proportion to the decrease in cell size (Galli and Morgan, 2016). We therefore examined the strength of SAC signaling in *Δnud-2* embryos at the 16- to 32-cell stage. The NEBD–anaphase onset interval in these multicellular embryos increased significantly from 143±3 s in controls to 174±8 s in *Δnud-2* embryos (Fig. 5C). The increase in mitotic duration was dependent on the SAC, as co-depletion of MAD-1 and MAD-2 in *Δnud-2* embryos reduced the NEBD–anaphase interval to that observed in controls (135±5 s). Thus, SAC signaling in early multicellular *Δnud-2* embryos is sufficiently robust to increase mitotic duration by ∼20% at the 16- to 32-cell stage. In control embryos and in embryos co-depleted of MAD-1 and MAD-2, just 1% and 5% of dividing cells exhibited lagging chromatin in anaphase, respectively, and this slightly increased to 10% in *Δnud-2* embryos (Fig. 5D). By contrast, co-depletion of MAD-1 and MAD-2 in *Δnud-2* embryos increased the frequency of lagging anaphase chromatin to 41% (Fig. 5D; Movie 5). Thus, in contrast to one-cell embryos, SAC signaling in early multicellular *Δnud-2* embryos significantly reduced the rate of chromosome mis-segregation. Overall, these results show that the SAC responds effectively to the subtle perturbation in kinetochore microtubule attachment caused by NUD-2 loss as early as the 16-cell stage. We conclude that SAC signaling keeps chromosome segregation errors at sub-lethal levels during embryogenesis when kinetochore dynein function is compromised. This likely explains why in RZZ-inhibited embryos, where the SAC is inoperable, loss of kinetochore dynein is lethal.

### The CENP-F-like proteins HCP-1 and HCP-2 recruit NUD-2 to kinetochores and the spindle independently of dynein-LIS-1 and RZZ–SPDL-1

**Fig. 6.**
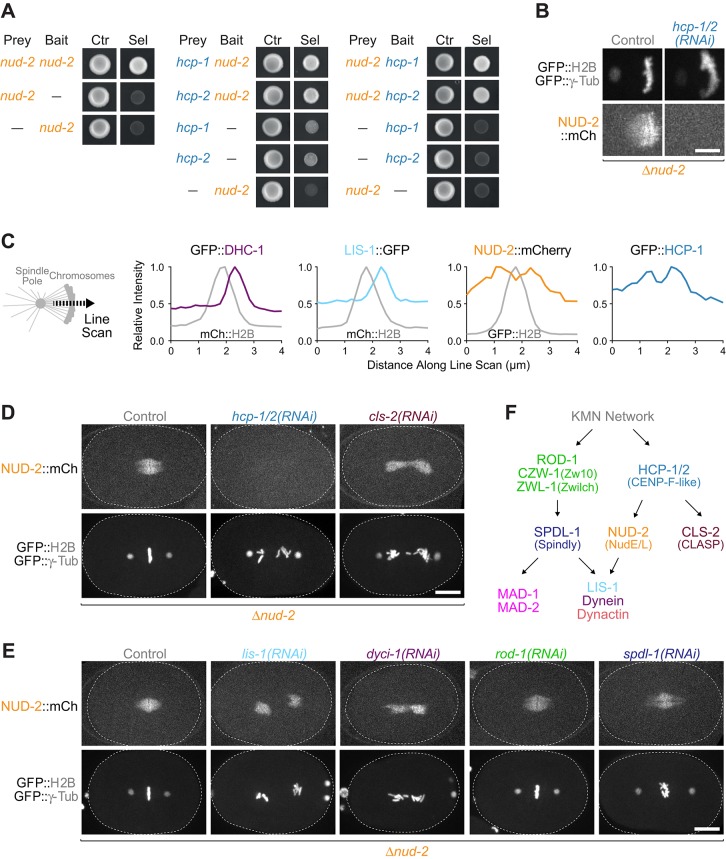
**HCP-1 and HCP-2 recruit NUD-2 to kinetochores and the spindle independently of dynein–LIS-1 and RZZ–SPDL-1.** (A) Yeast two-hybrid experiments showing that *nud-2* interacts with itself and with the paralogs *hcp-1* and *hcp-2*. Cells containing bait and prey plasmids grow on −Leu/−Trp plates (Ctr), while −Leu/−Trp/−His plates select for the interaction between bait and prey (Sel). (B) Stills from time-lapse sequences in monopolar AB cells (generated as described in Fig. 3A), showing that co-depletion of HCP-1 and HCP-2 [*hcp-1/2(RNAi)*] abolishes NUD-2::mCherry recruitment to kinetochores and the spindle. Scale bar: 5 µm. (C) Line scans across mitotic chromosomes in monopolar AB cells, showing that NUD-2::mCherry and GFP::HCP-1 are enriched on both the pole-proximal and pole-distal side, whereas GFP::DHC-1 and LIS-1::GFP accumulate exclusively on the pole-distal side. The graphs are representative of at least five monopolar spindles examined per condition. (D) Stills from time-lapse imaging sequences in one-cell embryos showing that NUD-2::mCherry localization requires HCP-1 and HCP-2 but not CLS-2. Scale bar: 10 µm. (E) Stills as in D showing that NUD-2::mCherry localizes independently of DYCI-1, LIS-1, ROD-1 and SPDL-1. Scale bar: 10 µm. (F) Summary of localization dependencies (arrows) in the two kinetochore pathways that recruit dynein for accurate chromosome segregation. The dotted line in D,E indicates the outline of the one-cell embryo.

Our results established that both NUD-2 and RZZ–SPDL-1 are essential for dynein function at kinetochores, and that NUD-2 contributes to dynein recruitment. To address the mechanism through which NUD-2 itself is recruited, we screened *nud-2* cDNA against a previously validated yeast two-hybrid library of *C. elegans* kinetochore components (Moyle et al., 2014). In addition to an interaction of *nud-2* with itself, we uncovered interactions between *nud-2* and the paralogs *hcp-1* and *hcp-2*, which share limited similarity with CENP-F (Fig. 6A) (Cheeseman et al., 2005; Moore et al., 1999). Co-depletion of HCP-1 and HCP-2 [*hcp-1/2(RNAi)*] abolished NUD-2::mCherry enrichment at kinetochores in the monopolar spindle assay as well as its localization to the spindle region in one-cell embryos (Fig. 6B,D; Movie 6). By contrast, depletion of the CLASP homolog CLS-2, whose recruitment depends on HCP-1 and HCP-2 (Cheeseman et al., 2005), did not affect NUD-2::mCherry localization (Fig. 6D). Furthermore, NUD-2::mCherry still localized to the chromosome/spindle region in one-cell embryos after depletion of LIS-1, the dynein intermediate chain DYCI-1, ROD-1 and SPDL-1 (Fig. 6E). Line scans performed on monopolar spindles showed that GFP::DHC-1 and LIS-1::GFP accumulated exclusively at sister kinetochores on the pole-distal side that were not occupied by spindle microtubules (unattached kinetochores) (Fig. 6C). By contrast, NUD-2::mCherry was enriched equally on pole-proximal and pole-distal kinetochores, and the same localization was observed for GFP::HCP-1 (Fig. 6C). Taken together, these results suggest that NUD-2 is recruited to kinetochores and the spindle through a direct interaction with the CENP-F-like proteins HCP-1 and HCP-2 and independently of dynein–LIS-1 and RZZ–SPDL-1 (Fig. 6F).

## DISCUSSION

Cytoplasmic dynein 1 is a multi-tasking motor with essential roles during interphase and mitosis, but how this complex molecular machine is regulated remains incompletely understood. A large amount of work *in vivo* and *in vitro* shows that Lis1 and its binding partners NudE/L are ubiquitous dynein co-factors that contribute to many processes including intracellular trafficking, centrosome positioning, nuclear migration, mitotic spindle assembly and chromosome segregation (Cianfrocco et al., 2015; Vallee et al., 2012). Surprisingly, we find that loss of the sole *C. elegans* NudE/L homolog NUD-2 is dispensable for the dynein-dependent pulling forces that position centrosomes and pronuclei in the zygote, as well as for mitotic spindle assembly. This contrasts with the absolute requirement for LIS-1, whose depletion results in a dynein-null phenotype, as documented by a previous study (Cockell et al., 2004). The observation that pronuclear migration in *Δnud-2* embryos shows enhanced sensitivity to a reduction in LIS-1 levels indicates that NUD-2 does contribute to dynein-dependent force generation but suggests that the effect of NUD-2 loss is buffered by LIS-1. The LIS-1 level-dependent penetrance of the *Δnud-2* phenotype parallels findings in fungi: the *A. nidulans* Lis1 homolog (*nudF*) is a multicopy suppressor of a *nudE* null mutant (Efimov, 2003), and in *S. cerevisiae* overexpression of the Lis1 homolog Pac1 suppresses a null mutant of *ndl1* (a NudE/L homolog) (Li et al., 2005). Similarly, spindle assembly defects after depletion of NudEL from *X. laevis* meiotic egg extracts could be rescued by addition of excess Lis1 (Wang and Zheng, 2011). Biochemical studies indicate that NudE/L tether Lis1 to dynein, thereby facilitating the interaction between Lis1 and the dynein motor domain that controls the affinity of dynein for microtubules (Huang et al., 2012; Liang et al., 2004; McKenney et al., 2010; Sasaki et al., 2000; Wang and Zheng, 2011; Wang et al., 2013; Zylkiewicz et al., 2011). Thus, increasing the concentration of Lis1 is predicted to make the Lis1–dynein interaction less dependent on NudE/L. In light of this tethering function for NudE/L, our results suggest that for most dynein- and LIS-1-dependent processes in the embryo, the LIS-1 concentration is high enough to allow sufficiently efficient binding to and regulation of dynein without NUD-2.

In contrast to other dynein functions, we show that dynein at kinetochores is significantly impaired by loss of NUD-2. In a quantitative pole tracking assay that monitors the ability of kinetochores to form load-bearing microtubule attachments, loss of NUD-2 fully recapitulates what is seen upon RZZ inhibition, which is known to remove dynein from kinetochores (Gassmann et al., 2008). Kinetochores without NUD-2, however, still recruit ∼50% of dynein. Kinetochores may require a higher ‘threshold’ of dynein activity relative to other subcellular sites where dynein would be predicted to also experience high load, such as the female nuclear envelope during pronuclear migration. Such a ‘threshold’ may relate to the affinity of individual motors for microtubules or to the number of motors engaged. The latter possibility is supported by the observation that reducing kinetochore dynein levels to ∼60% by partial RZZ depletion is sufficient to recapitulate the results seen upon complete dynein loss in the pole tracking assay. Finally, the need for NUD-2 at kinetochores may also reflect specialized regulation that is required for dynein to function in a high-density context.

The lagging anaphase chromatin observed after RZZ inhibition and NUD-2 loss can be attributed to the persistence of merotelic attachments, where a single sister kinetochore is simultaneously attached to microtubules from both spindle poles (Gassmann et al., 2008). In early prometaphase of cultured human cells, initial lateral interactions between kinetochores and microtubules have been proposed to rotate kinetochore pairs such that sister kinetochores face opposite poles prior to formation of end-coupled attachments, thus decreasing the probability for merotelic attachment (Magidson et al., 2015). Correct orientation of sister kinetochores prior to stable microtubule attachment is particularly important in *C. elegans*, whose large holocentric kinetochores invite merotely. When a kinetochore first captures a microtubule laterally, the minus-end-directed motility of dynein could provide the force that orients the kinetochore toward the spindle pole to which that microtubule is connected. Although NUD-2 loss mimics RZZ inhibition in the pole tracking assay, lagging anaphase chromatin is more prevalent after RZZ inhibition. A potential explanation for this difference is that, without NUD-2, lateral contacts between residual kinetochore dynein and microtubules may facilitate partial orientation of kinetochores without being long-lived enough to accelerate the integration of microtubule plus-ends into the KMN network (hence the negligible contribution to load-bearing attachment formation).

We show that NUD-2 is recruited to kinetochores and the spindle by a direct interaction with the CENP-F-like proteins HCP-1 and HCP-2. CENP-F and HCP-1 and HCP-2 are large coiled-coil proteins, but their sequences are highly divergent. The only obvious similarity is found within a tandem repeat (132 residues in HCP-1) that is present in both HCP-1 and CENP-F (Moore et al., 1999). Interestingly, the first instance of this repeat in CENP-F corresponds to a minimal NudEL-interacting fragment (Vergnolle and Taylor, 2007). Thus, the CENP-F homology region in HCP-1 and HCP-2 may participate in the interaction with NUD-2 that we identified in the yeast two-hybrid assay. We conclude that the CENP-F–NudE/L pathway for dynein recruitment is conserved in *C. elegans*.

We show that RZZ–SPDL-1 and NUD-2 do not depend on each other for localization. NUD-2 localization is also independent of dynein and LIS-1, further supporting the idea that NUD-2 acts as a kinetochore receptor for the motor. Importantly, NUD-2 loss not only reduces the kinetochore levels of dynein and LIS-1, but also the kinetochore levels of dynactin. This suggests that NUD-2, possibly through LIS-1, could locally stabilize the interaction between dynein and dynactin, in line with biochemical evidence from *X. laevis* egg extracts (Wang et al., 2013), *D. melanogaster* (Dix et al., 2013) and purified mammalian proteins (Baumbach et al., 2017). Our comparative analysis uncovered no evidence that RZZ–SPDL-1 and NUD-2 recruit functionally distinct dynein pools to kinetochores, nor that the two pathways engage with kinetochore dynein at different times during mitosis. Furthermore, biochemical studies indicate that Spindly and NudE/L interact with dynein through distinct binding sites (Gama et al., 2017; Wang and Zheng, 2011; Zylkiewicz et al., 2011), so it is likely that they can simultaneously bind to the same dynein motor. Given that NUD-2 cannot recruit a significant amount of dynein to kinetochores without RZZ–SPDL-1, we favor the idea that all of the dynein–Lis1 at kinetochores is integrated in a dynein–dynactin–Spindly complex, and that the NudE/L–dynein–Lis1 interaction contributes to the stability of this megadalton assembly.

Previous studies in human cultured cells and *D. melanogaster* have suggested that NudE/L are involved in the stripping of checkpoint proteins from microtubule-attached kinetochores (Vergnolle and Taylor, 2007; Wainman et al., 2009; Yan et al., 2003), implying that the resulting chronic SAC signaling should be deleterious for a developing organism. While we cannot rule out a SAC-silencing function for NUD-2 at the *C. elegans* kinetochore, both genetic and cellular assays strongly suggest that the main consequence of NUD-2 loss is SAC activation due to defects in kinetochore–microtubule attachment. The observation that the viability of *Δnud-*2 embryos is entirely dependent on the SAC implies that the penetrant lethality of RZZ inhibitions is a consequence of concurrent Mad1/Mad2 and dynein loss from kinetochores.

Our analysis of mitosis in early embryos illustrates that SAC signaling becomes more robust within the first few divisions. In *Δnud-2* embryos, there is no mitotic delay in the one-cell embryo, but mitotic duration increases by ∼20% at the 16–32 cell stage. A recent study in *C. elegans* uncovered that the SAC gains strength in proportion to the decrease in cell size (Galli and Morgan, 2016). In these experiments, the SAC was maximally activated by addition of a microtubule-depolymerizing drug and cells at the 16–32 cell stage exited mitosis through mitotic checkpoint slippage after arresting for >30 min (Galli and Morgan, 2016). Our results in *Δnud-2* embryos suggest that even minimal SAC activation, resulting in a mere 30 s extension of mitotic duration at the 16–32 cell stage, is sufficient to significantly decrease the frequency of chromosome segregation errors. Thus, the gradual gain in SAC strength during early embryogenesis has functional relevance under conditions that mildly perturb mitosis.

## MATERIALS AND METHODS

### Worm strains

Strains used in this study are listed in Table S1 and were maintained at 20°C on standard nematode growth medium (NGM) plates seeded with OP50 bacteria. A Mos1 transposon-based strategy (MosSCI) was used to generate a strain stably expressing NUD-2::mCherry::StrepTagII (Frøkjaer-Jensen et al., 2012, 2008). The transgene consisted of 3536 bp of the *nud-2* 5′ region, the *nud-2* open reading frame including all introns, *mCherry::StrepTagII* (with three artificial introns in *mCherry*), and 1090 bp of the *nud-2* 3′ region. The transgene was cloned into pCFJ151 for insertion on chromosome II (strain EG6429; locus ttTi5605), and integration was confirmed by performing PCR. Editing of the *lis-1* locus by CRISPR-Cas9 was performed with a single guide (sg)RNA targeting the sequence TTCGTCATCACAGGAAGTGTGG at the 3′ end of the *lis-1* gene and a repair template containing *gfp::StrepTagII* (with three artificial introns in *gfp*), flanked by 1618 bp of upstream sequence (measured from the Cas9 cut site), and by 1017 bp of downstream sequence (measured from the *lis-1* stop codon), followed by the *C. briggsae unc-119(+)* gene as a selectable marker and an additional 1498 bp of the *lis-1* 3′ region. Expression of sgRNA and Cas9 was performed according to published protocols (Dickinson and Goldstein, 2016), and correct tagging was confirmed by sequencing. Other transgenes and alleles were subsequently introduced by mating.

### RNA interference

For production of double-stranded RNA (dsRNA), oligonucleotides with tails containing T3 and T7 promoters were used to amplify regions from genomic N2 DNA (gDNA) or cDNA (Table S2). PCR products were purified (NucleoSpin Gel and PCR Clean-up, Macherey-Nagel) and used as templates for T3 and T7 transcription reactions (MEGAscript, Invitrogen). Transcription reactions were purified (NucleoSpin RNA Clean-up, Macherey-Nagel) and annealed in soaking buffer (3× soaking buffer is 32.7 mM Na_2_HPO_4_, 16.5 mM KH_2_PO_4_, 6.3 mM NaCl, 14.1 mM NH_4_Cl). dsRNAs were delivered by injecting L4 hermaphrodites. After injection, animals were incubated as follows before embryos were isolated for live-imaging: penetrant depletions, 48 h at 20°C; partial LIS-1 depletion (Fig. 1I,J), 24 h at 16°C; partial ROD-1 depletion (Fig. 3F,G), 24 h at 20°C; partial depletion of SPDL-1 and NDC-80 (Fig. 2D,E), 24 h at 16°C.

### Antibodies

Affinity-purified rabbit polyclonal antibodies against an N-terminal region of LIS-1 (residues 1–141) and a C-terminal region of DHC-1 (residues 3874–4074) were generated as described previously (Desai et al., 2003). In brief, GST::LIS-1_1-141_ and GST::DHC-1_3874-4074_ were expressed from pGEX6P1 in *Escherichia coli*, purified using glutathione resin (Thermo Scientific) and injected into rabbits (GeneCust). Serum was affinity purified on a HiTrap *N*-hydroxysuccinimide column (GE Healthcare) against covalently coupled LIS-1_1-141_ or DHC-1_3874-4074_.

### Immunoblotting

200 adult hermaphrodites were collected into 1 ml M9 buffer and processed for immunoblotting as described in detail in a previous study (Barbosa et al., 2017). The following primary antibodies were used: mouse monoclonal anti-α-tubulin B512 (Sigma, 1:5000), rabbit polyclonal anti-LIS-1 (made in-house, 1:2000), rabbit polyclonal anti-DHC-1 (made in-house, 1:1400), rabbit polyclonal anti-DYCI-1 (made in-house, 1:1000), rabbit polyclonal anti-DNC-1 (made in-house, 1:500) and rabbit polyclonal anti-DNC-2 (made in-house, 1:5000).

### Yeast two-hybrid analysis

Yeast two-hybrid analysis was performed according to the manufacturer's guidelines (Matchmaker, Invitrogen). Yeast containing bait (pGBKT7) and prey (pGADT7) vectors with the cDNAs of interest were plated on −Leu/−Trp/−His plates to select for interactions. Screening was performed using cDNAs encoding for full-length *C. elegans* kinetochore components (Moyle et al., 2014) and NUD-2.

### Embryonic viability

Embryonic viability assays were performed at 20°C. L4 hermaphrodites injected with dsRNA were grown for 40 h on NGM plates containing OP50 bacteria, single adults were placed on new mating plates (NGM plates with a small amount of OP50 bacteria), and removed 8 h later. The number of hatched and unhatched embryos on each plate was counted after further incubation for 16 h.

### Live-imaging of embryos and image analysis

Adult gravid hermaphrodite worms were dissected in a watch glass filled with Egg Salts medium (118 mM KCl, 3.4 mM MgCl_2_, 3.4 mM CaCl_2_ and 5 mM HEPES pH 7.4), and embryos were mounted on a fresh 2% agarose pad and covered with an 18 mm×18 mm coverslip (No. 1.5H, Marienfeld). Embryos co-expressing GFP::histone H2B and GFP::γ-tubulin for tracking of centrosomes and nuclei (Fig. 1F–H; Fig. 2C–G; Fig. 3G) were imaged on an Axio Observer microscope (Zeiss) equipped with an Orca Flash 4.0 camera (Hamamatsu), a Colibri.2 light source, and controlled by ZEN software (Zeiss). All other imaging was performed on a Nikon Eclipse Ti microscope coupled to an Andor Revolution XD spinning disk confocal system composed of an iXon Ultra 897 CCD camera (Andor Technology), a solid-state laser combiner (ALC-UVP 350i, Andor Technology), and a CSU-X1 confocal scanner (Yokogawa Electric Corporation), controlled by Andor IQ3 software (Andor Technology). All imaging was performed in temperature-controlled rooms kept at 20°C. Time-lapse sequences were processed and analyzed with Fiji software (Image J version 2.0.0-rc-56/1.51 h).

#### Pronuclear migration/centration, centrosome positioning, orientation of centrosome-centrosome axis, and centrosome–centrosome distance

Time-lapse sequences of GFP::histone H2B and GFP::γ-tubulin, consisting of 9×1 µm *z*-stacks for GFP fluorescence and one central differential interference contrast (DIC) image captured every 10 s, were recorded at 2×2 binning with a 63× NA 1.4 oil immersion objective (Zeiss) from the start of pronuclear migration (or just prior to NEBD for pole tracking) in the one-cell embryo until the onset of cytokinesis. Embryo length was defined as the distance between the outermost points of the egg shell visible in the DIC image. After maximum intensity projection of GFP *z*-stacks, the *x* and *y* coordinates of pronuclei and/or centrosomes were recorded over time using the MTrackJ plugin by manually clicking in the center of centrosomes and pronuclei.

#### Quantification of kinetochore levels on monopolar spindles

Time-lapse sequences of GFP-marked dynein, LIS-1 or dynactin, consisting of *z*-stacks of 9–12 planes with 1 µm separation captured every 10–20 s, were recorded at 1×1 binning with a 60× NA 1.4 oil immersion objective (Nikon) from 40–50 s prior to NEBD until chromosome decondensation in the AB cell of the two-cell embryo. The kinetochore signal was measured at between 180 and 200 s after NEBD in a maximum intensity projection of the z-stack. The top 20 local maxima on chromosomes were identified by using the ‘Find Maxima’ function, and the values were averaged. The mean fluorescence intensity of the spindle background close to the chromosomal region was measured and subtracted from the kinetochore signal.

#### Quantification of kinetochore levels in metaphase one-cell embryos

Time-lapse sequences of GFP and mCherry fusions, consisting of *z*-stacks of nine planes with 1 µm separation captured every 10 s, were recorded at 1×1 binning with a 60× NA 1.4 oil immersion objective (Nikon) from 40–50 s prior to NEBD until chromosome decondensation. Kinetochore fluorescence intensities were determined at four to six frames before the onset of sister chromatid separation, when chromosomes were aligned at the metaphase plate, as described previously (Moyle et al., 2014). Briefly, a region was drawn around chromosomes (marked by GFP- or mCherry-tagged histone H2B), and the integrated fluorescence intensity was measured for the kinetochore component (mCherry::ROD-1, GFP::SPDL-1 or GFP::MAD-1). The chromosomal region was expanded by 1–2 pixels on all sides, and the difference in integrated intensity between the expanded and chromosomal region was used to define the background intensity. The final integrated intensity for the chromosomal region was calculated by subtracting the background intensity.

#### Quantification of kinetochore levels over time in one-cell embryos

Time-lapse sequences of mCherry::ROD-1 and GFP::histone H2B, and GFP::SPDL-1 and mCherry::histone H2B were recorded as described above for the quantification of kinetochore levels in metaphase. In each frame, the ‘Find Maxima’ function in Fiji was used to identify the top 15 local maxima in a region containing the mitotic chromosomes, and the values were averaged. The averaged intensity of the top 15 local maxima of an adjacent cytoplasmic region of similar size was subtracted as background.

#### Quantification of lagging anaphase chromatin

Time-lapse sequences of GFP::histone H2B and GFP::γ-tubulin, consisting of *z*-stacks of 11 planes with 1 µm separation (one-cell embryos in Fig. 2B) or *z*-stacks of 22 planes with 1 µm separation (multicellular embryos in Fig. 5B,D) captured every 10 s, were recorded at 1×1 binning with a 100× NA 1.45 oil immersion objective (Nikon). Lagging anaphase chromatin was defined as visible threads of GFP::histone H2B signal between separating chromatid masses at two to four frames after the onset of sister chromatid separation.

### Statistical analysis

Values in figures and text are reported as mean±95% c.i. Statistical analysis was performed with GraphPad Prism 7.0 software. The type of statistical analysis (*t*-test or one-way ANOVA/Bonferroni's multiple comparison test) is indicated in the figure legends. Differences were considered significant at *P*<0.05.

## Supplementary Material

Supplementary information
