# Supplementary material for: NudE regulates dynein at kinetochores but is dispensable for other dynein functions in the C. elegans early embryo
Source: J Cell Sci. 2018 Jan 1;131(1):jcs212159. doi: 10.1242/jcs.212159 (PMC5818066; doi:10.1242/jcs.212159)
Supplement: Supplementary information [file joces-131-212159-s1.pdf]

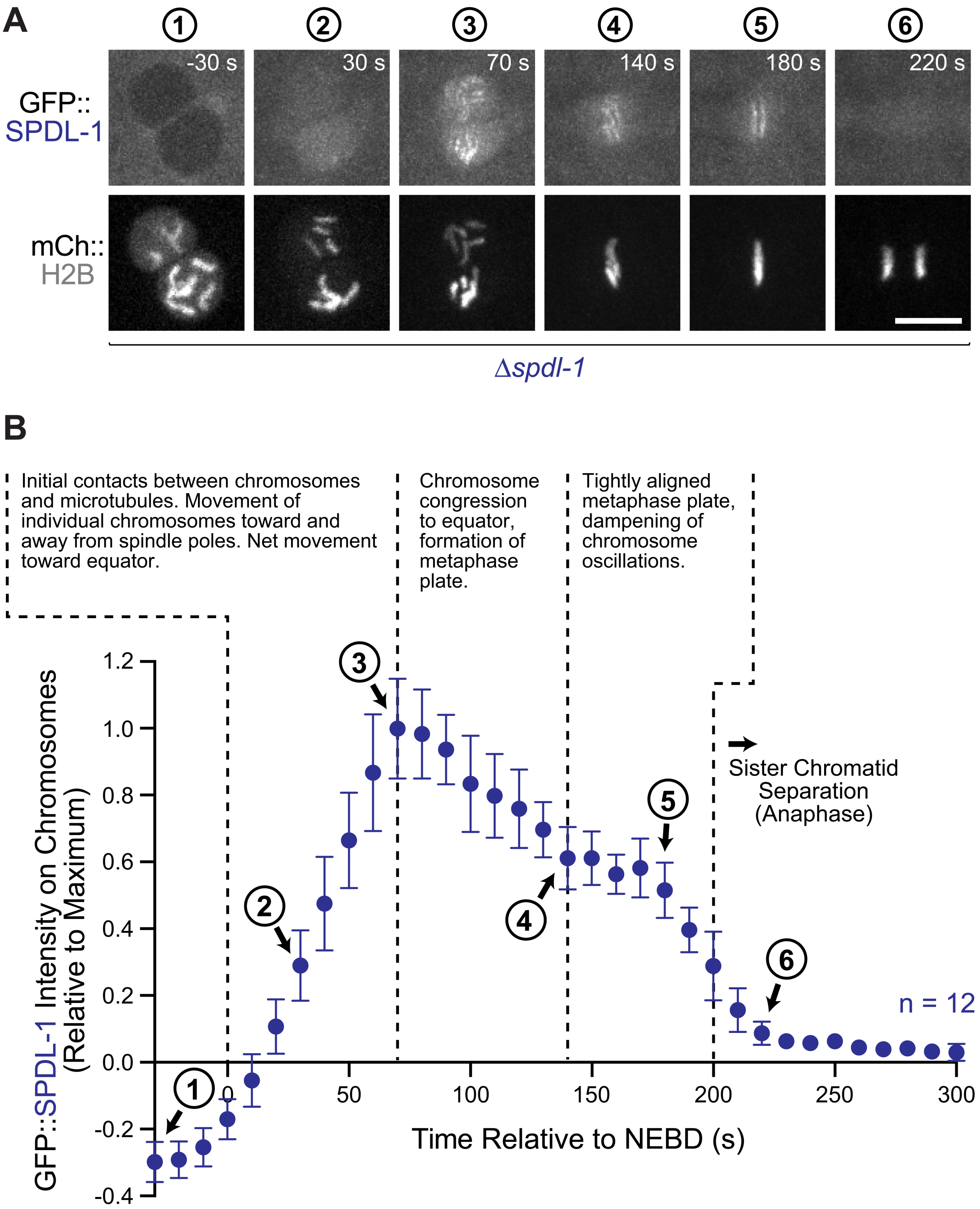

Journal of Cell Science • Supplementary information

Figure S1: Kinetochore levels of Spindly^{SPDL-1} are inversely correlated with chromosome congression.

(A) Stills from a time-lapse sequence in a one-cell embryo co-expressing GFP::Spindly^{SPDL-1} and mCherry::histone H2B. Numbers on top refer to the time points highlighted in the graph shown in *(B)*. Scale bar, 10 µm.

(B) Quantification of GFP::Spindly^{SPDL-1} localization dynamics in the first embryonic division, using time-lapse sequences as shown in *(A)*. Images were acquired at 10 s intervals and the fluorescence intensity of GFP on chromosomes was determined for each time point. Traces were aligned relative to NEBD and normalized to the maximum average signal. The number *n* of embryos imaged is indicated. Error bars represent the SEM with a 95 % confidence interval.

Journal of Cell Science • Supplementary information

Table S1: *C. elegans* strains

Strain Name	Genotype
N2	*wild type (ancestral N2 Bristol)*
GCP24	*ltSi426[pDC192; Prod-1::mCherry::rod-1::rod-1 3'UTR; cb-unc-119(+)] II; unc-119(ed3) III?; ruIs32[pAZ132; Ppie-1::GFP::his-58; cb-unc-119(+)] III; ddIs6[Ppie-1::GFP::tbg-1; cb-unc-119(+)] V **
GCP39	*nud-2(ok949) I; unc-119(ed3) III?; ruIs32[pAZ132; Ppie-1::GFP::his-58; cb-unc-119(+)] III; ddIs6[Ppie-1::GFP::tbg-1; cb-unc-119(+)] V **
GCP66	*nud-2(ok949) I* (outcrossed 6x with N2)
GCP96	*nud-2(ok949) I; unc-119(ed3) III?; prtSi37[pRG200;Pnud-2::nud-2::mCherry::StrepTagII::nud-2 3'UTR; cb-unc-119(+)] II; ruIs32[pAZ132; Ppie-1::GFP::his-58; cb-unc-119(+)] III; ddIs6 [Ppie-1::GFP::tbg-1; cb-unc-119(+)] V **
GCP146	*nud-2(ok949) I; unc-119(ed3) III?; orIs17[Pdhc-1::GFP::dhc-1, cb-unc-119(+)]; ltIs37[pAA64; Ppie-1::mCherry::his-58; cb-unc-119(+)] IV **
GCP163	*prt104[lis-1::GFP::1 kb lis-1 3' UTR::cb-unc-119(+)]; unc-119(ed3) III*
GCP186	*prt104[lis-1::GFP::1 kb lis-1 3' UTR::cb-unc-119(+)]; unc-119(ed3) III?; itIs37[pAA64; Ppie-1::mCherry::his-58 cb-unc-119(+)] IV **
GCP211	*nud-2(ok949) I; prt104[lis-1::GFP::1 kb lis-1 3' UTR::cb-unc-119(+)]; unc-119(ed3) III?; itIs37[pAA64; Ppie-1::mCherry::his-58 cb-unc-119(+)] IV **
GCP326	*unc-119(ed3) III?; ruIs32[pAZ132; Ppie-1::GFP::his-58; cb-unc-119(+)] III; ddIs6[Ppie-1::GFP::tbg-1; cb-unc-119(+)] V; weIs21[pJA138; Ppie-1::mCherry::β-tubulin] IV*
GCP418	*nud-2(ok949) I; spdl-1(ok1515) II; jzIs41[pRK177; Ppie-1::GFP::spdl-1; cb-unc-119(+)]; unc-119(ed3) III?; itIs37[pAA64; Ppie-1::mCherry::his-58 cb-unc-119(+)] IV **
GCP448	*nud-2(ok949) I; ltSi608[pOD1583/pMM30; Pmdf-1::GFP::mdf1::mdf-1 3'UTR; cb-unc-119(+)] II; mdf-1(gk2) V; unc-119(ed3)III?; ltIs37[pAA64; Ppie-1::mCherry::his-58; cb-unc-119(+)] IV **
GCP459	*nud-2(ok949) I; ltSi426[pDC192; Prod-1::mCherry::rod-1::rod-1 3'UTR; cb-unc-119(+)] II; unc-119(ed3) III?; ruIs32[pAZ132; Ppie-1::GFP::his-58; cb-unc-119(+)] III; ddIs6[Ppie-1::GFP::tbg-1; cb-unc-119(+)] V **
GCP505	*nud-2(ok949) I; unc-119(ed3) III?; ruIs32[pAZ132; Ppie-1::GFP::his-58; unc-119(+)] III; ddIs6 [GFP::tbg-1; cb-unc-119(+)] V; weIs21[pJA138; Ppie-1::mCherry::β-tubulin] IV **
GCP576	*nud-2(ok949) I; unc-119(ed3) III?; tjIs11[Ppie-1::GFP::dnc-2; unc-119 (+)] **
OD7	*unc-119(ed3) III; ltIs3[pIC31; Ppie-1::GFP-TEV-STag::hcp-1; cb-unc-119(+)]*
OD56	*unc-119(ed3) III; ltIs37[pAA64; Ppie-1::mCherry::his-58; cb-unc-119 (+)] IV*
OD203	*unc-119(ed3) III?; orIs17[Pdhc-1::GFP::dhc-1; cb-unc-119(+)]; ltIs37[pAA64; Ppie-1::mCherry::his-58; cb-unc-119(+)] IV **
OD204	*unc-119(ed3) III?; tjIs11[Ppie-1::GFP::dnc-2; cb-unc-119(+)]; ltIs122[pAA64; Ppie-1::mCherry::his-58; cb-unc-119(+)] IV **
OD1209	*ltSi608[pOD1583/pMM30; Pmdf-1::GFP::mdf1::mdf-1 3'UTR; cb-unc-119(+)] II; mdf-1(gk2) V; unc-119(ed3) III?; ltIs37[pAA64; Ppie-1::mCherry::his-58; cb-unc-119(+)] IV **
RQ283	*unc-119(ed3) III?; jzIs41[pRK177; Ppie-1::GFP::spdl-1; cb-unc-119(+)]; spdl-1(ok1515) II; itIs37[pAA64; Ppie-1::mCherry::his-58; cb-unc-119(+)] IV **
TH32	*unc-119(ed3) III?; ruIs32[pAZ132; Ppie-1::gfp::his-58; cb-unc-119(+)] III; ddIs6[Ppie-1:gfp::tbg-1; cb-unc-119(+)] V*

* *unc-119(ed3) III* was present in parental strains, but these strains have not been sequenced to determine whether the *unc-119* gene still contains the *ed3* mutation.

Journal of Cell Science • Supplementary information

Table S2: Oligos for double-stranded RNA production

Gene ID	Gene Name	Oligo 1 (T3 promoter)	Oligo 2 (T7 promoter)	Template
C06A8.5	*spdl-1*	AATTAACCCTCACTAAAGGAACGTTACCCGAATGACCAC	TAATACGACTCACTATAGGCCTAATTGAGGCATGGGTTC	cDNA
C17H12.1	*dyci-1*	AATTAACCCTCACTAAAGGGGCAACTTTCGACTTGTCA	TAATACGACTCACTATAGGCTCTTGCAAAGTAATCAG	cDNA
C50F4.11	*mdf-1*	AATTAACCCTCACTAAAGGAGCATCCTCAAGTCGTTCGT	TAATACGACTCACTATAGGAAGCGAAGTTGGCTGAAAAA	gDNA
F55G1.4	*rod-1*	AATTAACCCTCACTAAAGGAATGCAAATCTTTTTGGATGGG	TAATACGACTCACTATAGGCATCGACGAATTTGATTCGATC	cDNA
F59E12.2	*zyg-1*	AATTAACCCTCACTAAAGGTGGACGGAAATTCAAACGAT	TAATACGACTCACTATAGGAACGAAATTCCCTTGAGCTG	cDNA
R107.6	*cls-2*	AATTAACCCTCACTAAAGGGATGGTTGCCTCAAGCTCTC	TAATACGACTCACTATAGGGCCAGGAAGACTGGAACAAA	gDNA
T03F6.5	*lis-1*	AATTAACCCTCACTAAAGGTCGGAGAGGCAAAAAGAAGA	TAATACGACTCACTATAGGCTCGAACCCAATTTTCGTGT	cDNA
T06E4.1	*hcp-2*	AATTAACCCTCACTAAAGGTCGTTGTCTCCAATTCCACA	TAATACGACTCACTATAGGTCTCGGAAAGGAATCGAAAA	gDNA
W01B6.9	*ndc-80*	AATTAACCCTCACTAAAGGCCCCAGTCTGAGTCAACCTC	TAATACGACTCACTATAGGCCAACTCGCTTTGAATTTCC	gDNA
Y54G9A.6	*bub-3*	AATTAACCCTCACTAAAGGGTCCCGTTTCCCCCATTTGT	TAATACGACTCACTATAGGTGTGGCGAATGGTGATCGAA	cDNA
Y69A2AR.30	*mdf-2*	AATTAACCCTCACTAAAGGACGGATGTAAAGACACAAAACG	TAATACGACTCACTATAGGGTGAACTGACGTCGAGAATGAG	cDNA
ZC328.4	*san-1*	AATTAACCCTCACTAAAGGTGACGCAGAAGATGATTGGGA	TAATACGACTCACTATAGGTGCGCAATTAGTTCGAGCAAG	cDNA
ZK1055.1	*hcp-1*	AATTAACCCTCACTAAAGGAGATCGCGCTGAAGACTTTC	TAATACGACTCACTATAGGAAACCGAGTCGCCATTTTC	gDNA

Journal of Cell Science • Supplementary information

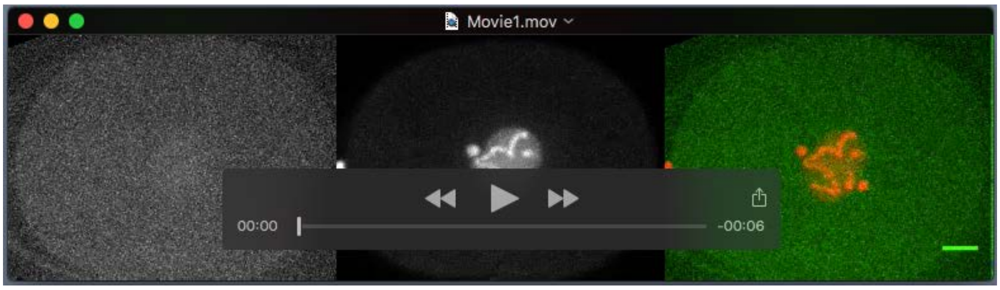

Movie 1: Localization of NUD-2::mCherry during the first embryonic division.

The embryo expresses transgene-encoded NUD-2::mCherry in a *Δnud-2* background, as well as GFP::histone H2B and GFP::γ-tubulin. Anterior side is on the left. Time lapse is 10 s and playback speed is 6 frames per second. Scale bar, 5 µm.

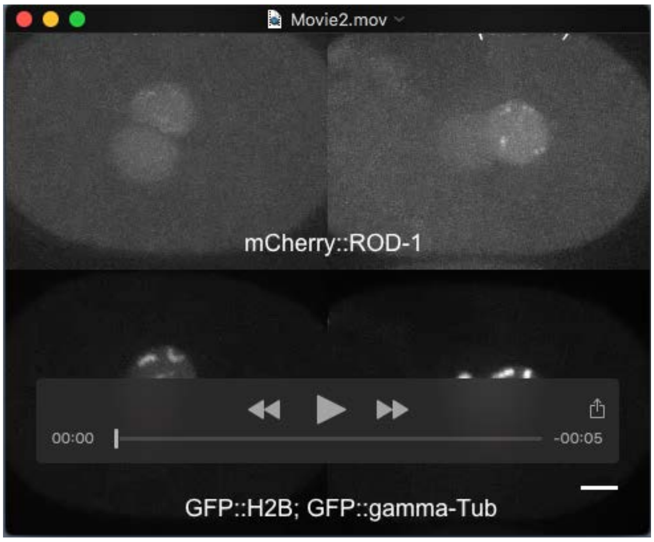

Movie 2: Increased levels of mCherry::ROD-1 at kinetochores in the one-cell *Δnud2* embryo.

Embryos express transgene-encoded mCherry::ROD-1, as well as GFP::histone H2B and GFP::γ-tubulin. Anterior side is on the left. Time lapse is 10 s and playback speed is 6 frames per second. Scale bar, 5 µm.

Journal of Cell Science • Supplementary information

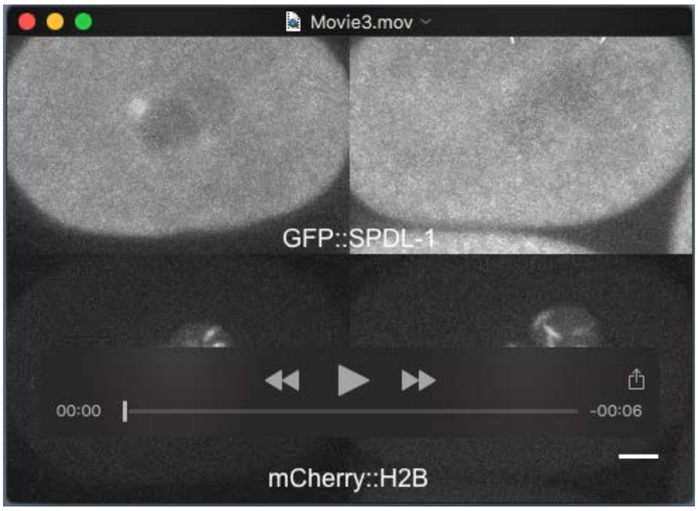

Movie 3: Increased levels of GFP::Spindly^{SPDL-1} at kinetochores in the one-cell *Δ nud2* embryo.

Embryos express transgene-encoded GFP::Spindly^{SPDL-1} and mCherry::histone H2B in a *spdl-1* null background. Anterior side is on the left. Time lapse is 10 s and playback speed is 6 frames per second. Scale bar, 5 μm.

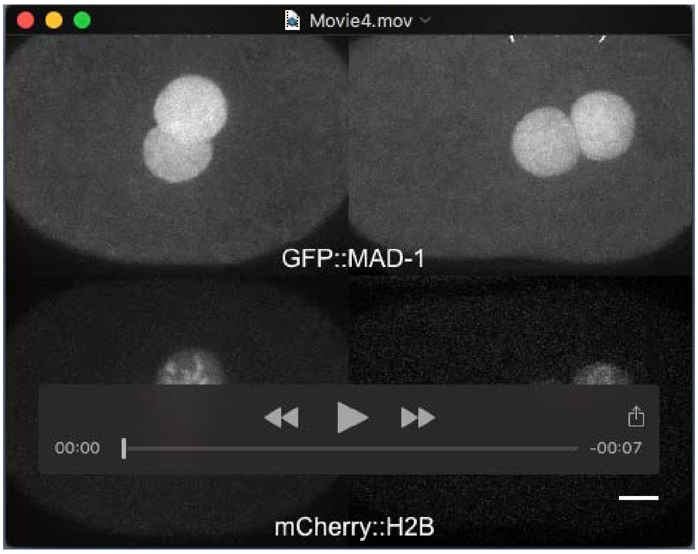

Movie 4: Increased levels of GFP::MAD-1 at kinetochores in the one-cell *Δnud2* embryo.

Embryos express transgene-encoded GFP::MAD-1 and mCherry::histone H2B in a *mad-1* null background. Anterior side is on the left. Time lapse is 10 s and playback speed is 6 frames per second. Scale bar, 5 μm.

Journal of Cell Science • Supplementary information

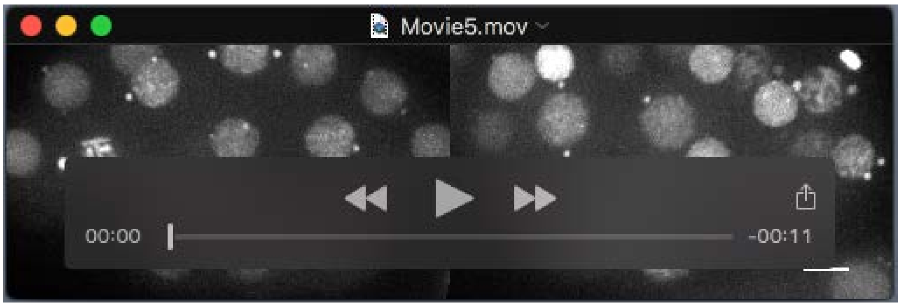

Movie 5: Co-depletion of MAD-1 and MAD-2 in multicellular *Δnud2* embryos results in severe chromosome mis-segregation.

Embryos (~32-cell stage) express GFP::histone H2B and GFP::γ-tubulin. Time lapse is 10 s and playback speed is 6 frames per second. Scale bar, 5 μm.

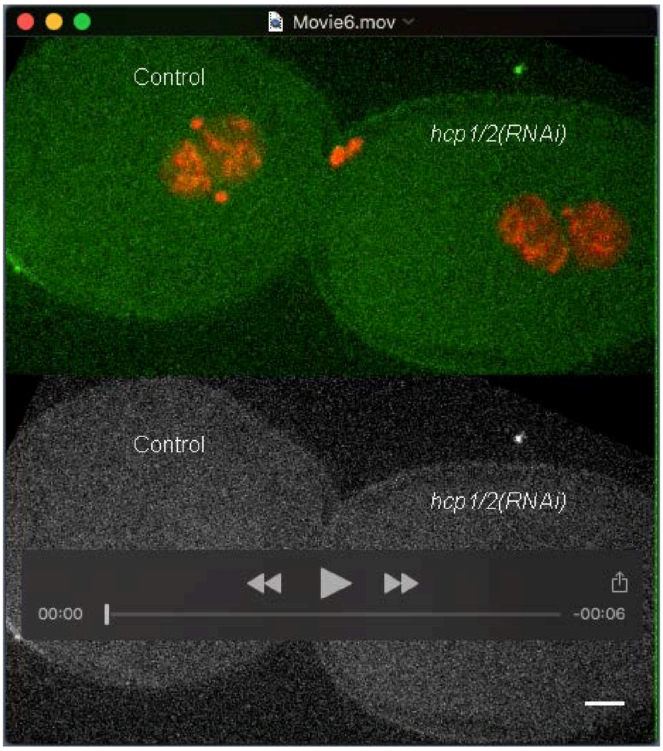

Movie 6: Co-depletion of HCP-1 and HCP-2 delocalizes NUD-2::mCherry.

Embryos express transgene-encoded NUD-2::mCherry in a *Δnud-2* background, as well as GFP::histone H2B and GFP::γ-tubulin. Anterior side is on the left. Time lapse is 10 s and playback speed is 6 frames per second. Scale bar, 5 μm.
